# In vitro comparison of cyclic fatigue resistance of TruNatomy in single and double curvature canals compared with different nickel-titanium rotary instruments

**DOI:** 10.1186/s12903-020-1027-7

**Published:** 2020-02-04

**Authors:** Amr M. Elnaghy, Shaymaa E. Elsaka, Ayman O. Mandorah

**Affiliations:** 1 Department of Endodontics, Faculty of Dentistry, Mansoura University, Mansoura, PC 35516 Egypt; 2 Department of Dental Biomaterials, Faculty of Dentistry, Mansoura University, Mansoura, Egypt; 3 Department of Restorative Science, Alfarabi Private College for Dentistry and Nursing, Jeddah, Saudi Arabia; 4 Department of Restorative and Dental Materials, Faculty of Dentistry, Taif University, Taif, Saudi Arabia

**Keywords:** Cyclic fatigue, Double curvature, Heat-treated alloy, TruNatomy, Weibull analysis

## Abstract

**Background:**

The purpose of this study was to compare the cyclic fatigue resistance of newly developed TruNatomy instruments (TRN) in single and double (S-shaped) curvature canals with HyFlex CM (HCM), Vortex Blue (VB) and RaCe (RC) instruments.

**Methods:**

Size 20/.04 taper and size 25/0.04 of HCM, VB and RC were used. For TRN instruments, size 20/.04 taper (small) and size 26/.04 taper (prime) were used. The instruments were tested in artificial canals with double curvature (coronal curve; 60° curvature, 5 mm radius and apical curve; 70° curvature and 2 mm radius) and single curvature (60° curvature, 5 mm radius). The number of cycles to failure (NCF) was recorded. Data were statistically analyzed by Kruskal-Wallis and Dunn’s multiple comparison tests. Weibull analysis was performed on NCF data. Statistical significant was set at *p* < 0.05.

**Results:**

TRN and HCM revealed higher NCF compared with the other instruments for both tested sizes in single and double curvature canals (*p* < 0.05). TRN and HCM showed no statistically significant difference in the NCF (*p* > 0.05). The probability of survival was higher for HCM and TRN instruments than VB and RC instruments.

**Conclusions:**

HCM and TRN instruments were more resistant to cyclic fatigue than VB and RC instruments in single and double curvature canals. HCM and TRN instruments were anticipated to survive with higher number of cycles than the other tested instruments. RC instrument had the lowest fatigue resistance than the other instruments.

## Background

In recent years, various thermomechanical treatments of nickel-titanium (NiTi) alloys have been developed to enhance the mechanical properties and clinical performance of NiTi rotary instruments [1–3]. Thermal processing is considered one of the most effective approaches to modify the transition temperatures of NiTi alloys [1, 4]. It has been reported that alterations in the transformation behavior through heat treatment were effectual enhancing the flexibility and fatigue resistance of NiTi endodontic instruments [2, 5–7].

HyFlex CM instruments (HCM) (Coltène-Whaledent, Altstätten, Switzerland) are manufactured using a distinctive method to control material memory through a complex heating and cooling treatment [4]. It has been reported that this heat-treated alloy, together with the distinctive design characteristics of the instruments, enhances the flexibility of the instruments but without the shape memory of conventional superelastic forms of NiTi alloy [8–10]. Consequently, the controlled memory (CM) NiTi instruments allow better maintenance of the original canal curvature and improved the efficacy of instruments in root canal treatment [9, 11].

Vortex Blue instrument (VB) (Dentsply Sirona, Ballaigues, Switzerland) is another system that controls the shape memory of NiTi alloy which improved the fatigue resistance of the instrument [2, 7, 12]. The manufacturing of VB is based on a proprietary Blue technology that forms a blue oxide layer on the surface of the instrument. The blue titanium oxide layer of VB is supposed to enhance the cutting efficiency and wear resistance of the instrument [1, 2, 7, 13]. The Blue technology allows the instrument to reach the martensitic phase during clinical treatment that enhances the fatigue resistance compared with the other instruments which are mainly in the more rigid austenitic phase [14]. RaCe (RC; FKG Dentaire, La Chaux-de-Fonds, Switzerland) is an electropolished instrument that is manufactured from a conventional NiTi alloy [15]. The RC instruments have sharp cutting edges to enhance efficiency and alternating cutting edges to remove screwing [16]. Fracture of NiTi rotary instruments is occurred by either flexural or torsional fatigue [17, 18]. Most of the teeth have curved canals, not only in one but in several orientations and different planes. Double curvatures (S-shaped) root canals can be troublesome and challenging during root canal instrumentation [3]. It has been reported that the fatigue resistance of NiTi instruments in double curvature canals was lower than in single curvature canals [3, 19]. Decreasing the probability of NiTi rotary instruments fracture has been one of the main aims of manufacturers in order to enhance safety by using innovative manufacturing processes [20].

Recently, TruNatomy instruments (TRN) (Dentsply Sirona) has been developed as a novel type of heat-treated NiTi instrument with a special design. The TRN shaping instruments are provided in three different sizes which are small (size 20/.04 taper)**,** prime (size 26/.04 taper) and medium (size 36/.03 taper). It has been claimed by the manufacturer that the three shaping instruments of TRN provide a slim shaping which enhances the debridement due to more space is available by this unique design of the instrument. The slim NiTi wire design is 0.8 mm instead of up to 1.2 mm of the most other variable tapered instruments. The TRN instruments are off-centred The TRN instruments are off-centred parallelogram cross-section design [21]. It was manufactured by using a special NiTi heat-treated wire that supposed to enhance the flexibility of the instrument. It had been reported that the TRN instruments preserve the structural dentine and tooth integrity due to instrument geometry, regressive tapers and the slim design, along with the heat-treatment of the NiTi alloy [21, 22]. There is no data available on the fatigue resistance of TRN instruments. Consequently, the purpose of the present study was to compare the resistance to cyclic fatigue of newly developed TRN in artificial single and double (S-shaped) curvature canals with other NiTi rotary instruments.

## Methods

### Cyclic fatigue resistance

**Fig. 1:**
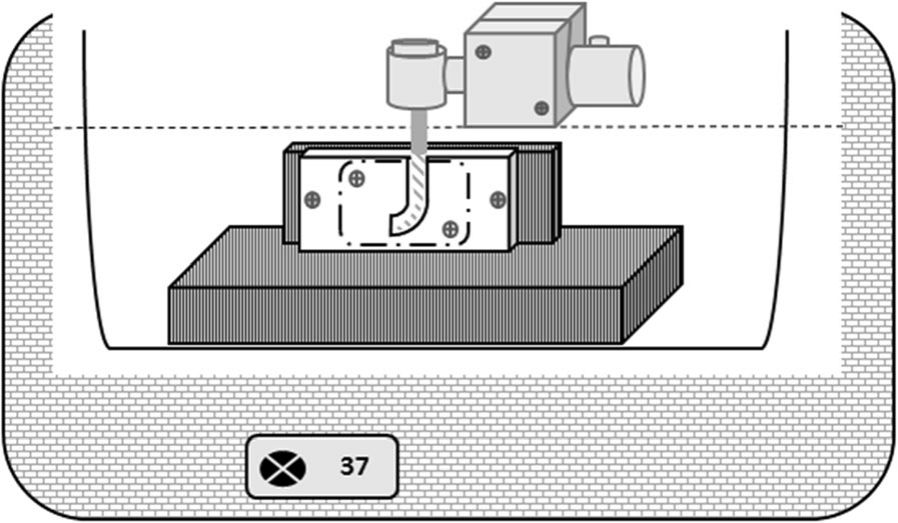
Schematic illustration of cyclic fatigue testing device. The instruments were immersed in saline during testing at 37 ± 1 °C. The handpiece was held by a jig during testing

Four NiTi rotary instruments with two different sizes were selected for this study. Size 20/.04 taper and size 25/.04 of HCM, VB and RC were used. For TRN instruments, size 20/.04 taper (small) and size 26/.04 taper (prime) were used. A total of 120 instruments for each system were divided into two groups (*n* = 60/group) according to the size of the instrument. Then, each group subdivided into two subgroups (*n* = 30/group) according to the curvature of the canals (single or double). All instruments were subjected to cyclic fatigue testing using a custom-made stainless steel block containing double curvature canal (60° coronal curvature with a 5 mm radius and an apical 70° curvature with a 2 mm radius) and single curvature canal (60° curvature with a 5 mm radius) (Fig. 1) which had been used in previous studies [3, 12, 19]. The instruments were operated in the X-Smart motor (Dentsply Sirona, York, PA, USA) with each specified speed and torque according to the manufacturer. The cyclic fatigue test was performed with instruments immersed in saline at 37 ± 1 °C. For each instrument, the number of cycles to fracture (NCF) was calculated. The NCF was calculated by multiplying the time recorded in minutes by the recommended motor speed. The fracture length of each instrument fragment was measured. The fractured surface of representative specimens was examined using a scanning electron microscope (SEM) (Stereoscan 260; Cambridge Instruments, Cambridge, UK).

### Statistical analysis

The normality of the data distribution and the homogeneity of variances were tested using Kolmogorov–Smirnov and Levene tests. The data showed a nonparametric distribution. The NCF and the fractured fragment length data were statistically analyzed by Kruskal-Wallis and Dunn’s multiple comparison tests. The Mann-Whitney test was performed between the data of different sizes of the same instrument. The statistical analysis was performed by using SPSS 20 software (SPSS Inc., Chicago, IL, USA). The level of significance was set at *p* < 0.05. Weibull analysis was performed on NCF data [23–25].

## Results

Table 1 showed the data of NCF and fragment length for TRN, HCM, VB and RC instruments. All the instruments showed significantly higher NCF values for size 20/.04 than size 25/.04 in the same curvature (*p* < 0.05). The instruments fractured first in the apical curvature then in the coronal curvature in the double curved canal. TRN and HCM revealed significantly higher NCF compared with the other instruments for both tested sizes in single and double curvature canals (*p* < 0.05). VB had significantly higher NCF than RC for both tested sizes (*p* < 0.05). TRN and HCM showed no significant difference in the NCF (*p* > 0.05). The RC instruments had significantly the lowest NCF values compared with other instruments (*p* < 0.05). There was no significant difference in the fragment length value in all groups (*p* > 0.05). The mean length of broken fragments in single and coronal curvature canals was significantly longer than that of fragments in the apical double curvature canal (*p* < 0.05).

**Table 1:** Mean ± standard deviations, median of number of cycles to failure (NCF), and length (mm) of fractured fragments (FL) of tested instruments

Groups	Single curvature			Double curvature					
				Apical curvature			Coronal curvature		
	NCF		FL	NCF		FL	NCF		FL
	Mean ± SD	Median	Mean ± SD	Mean ± SD	Median	Mean ± SD	Mean ± SD	Median	Mean ± SD
TRN									
20/.04	1327.4 ± 100.7^A^	1317	5.17 ± 0.14	619.2 ± 52.3^A^	629	2.12 ± 0.07	719.6 ± 79.4^A^	716	5.07 ± 0.15
26/.04	1238.8 ± 106.7^a^	1277	5.17 ± 0.13	532.8 ± 51.9^a^	523	2.12 ± 0.06	609.2 ± 46.5^a^	614	5.05 ± 0.14
HCM									
20/.04	1388.1 ± 101.9^A^	1362	5.15 ± 0.11	623.9 ± 57.4^A^	629	2.11 ± 0.09	737.8 ± 73.1^A^	715	5.08 ± 0.19
25/.04	1296.3 ± 80.3^a^	1265	5.18 ± 0.18	542.6 ± 52.4^a^	544	2.13 ± 0.17	618.6 ± 51.4^a^	621	5.07 ± 0.19
VB									
20/.04	704.3 ± 55.1^B^	712	5.14 ± 0.21	447.3 ± 71.7^B^	430	2.16 ± 0.11	540.4 ± 55.3^B^	549	5.04 ± 0.25
25/.04	529.5 ± 56.8^b^	516	5.16 ± 0.21	290.2 ± 33.5^b^	290	2.15 ± 0.17	358.4 ± 44.1^b^	351	5.08 ± 0.22
RC									
20/.04	232.3 ± 29.5^C^	230	5.13 ± 0.07	122.5 ± 20.7^C^	125	2.13 ± 0.17	140.1 ± 21.1^C^	140	5.04 ± 0.15
25/.04	215.9 ± 22.4^c^	215	5.14 ± 0.08	93.1 ± 12.1^c^	95	2.14 ± 0.17	127.7 ± 15.1^c^	126	5.03 ± 0.18

Different superscript uppercase letter (column) for instrument size 20/.04 and lowercase letter (column) for sizes 25/.04 and 26/.04 indicate statistically significant difference (*P* < 0.05)

**Fig. 2:**
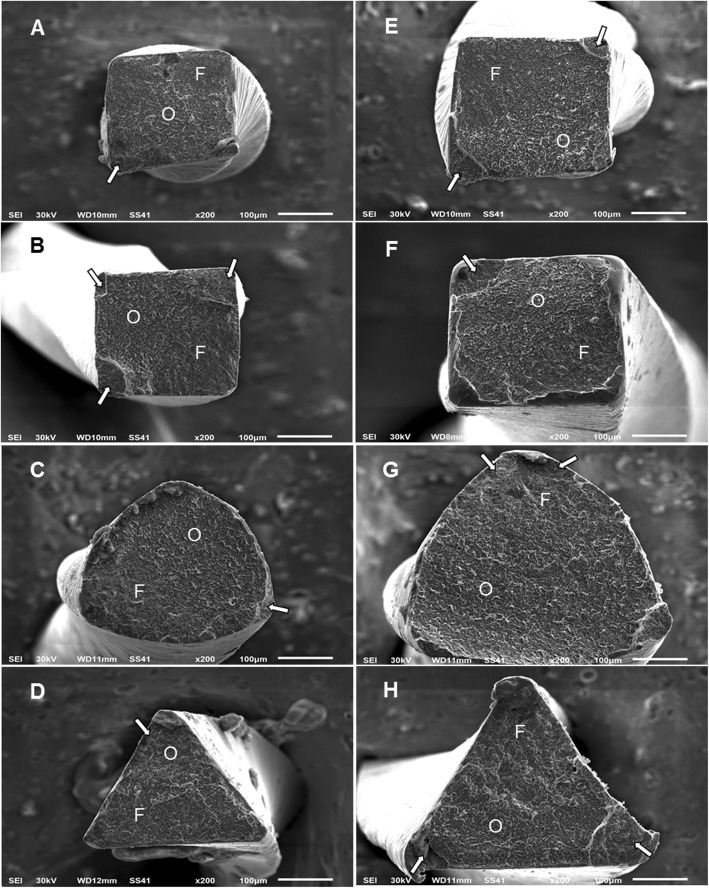
SEM images (× 200) of fractured fragments in double curvature canals. Fracture of instruments in apical curvature (**a-d**) and coronal curvature (**e-h**). (**a**, **e**) TRN, (**b**, **f**) HCM, (**c**, **g**) VB and (**d**, **h**) RC; respectively. The following features could be observed: the origin of the crack (arrow), fatigue zone (**f**), and overload fast fracture zone (**o**)

The SEM images of broken segments in the double curvature canal are presented in Fig. 2. All the instruments showed the ductile fracture of cyclic fatigue failure. TRN instrument showed a parallelogram cross-section design while HCM instruments revealed a square cross-section design (Fig. 2a and b; respectively). On the other hand, VB and RC instruments revealed triangular cross-section design (Fig. 2c and d; respectively).

**Fig. 3:**
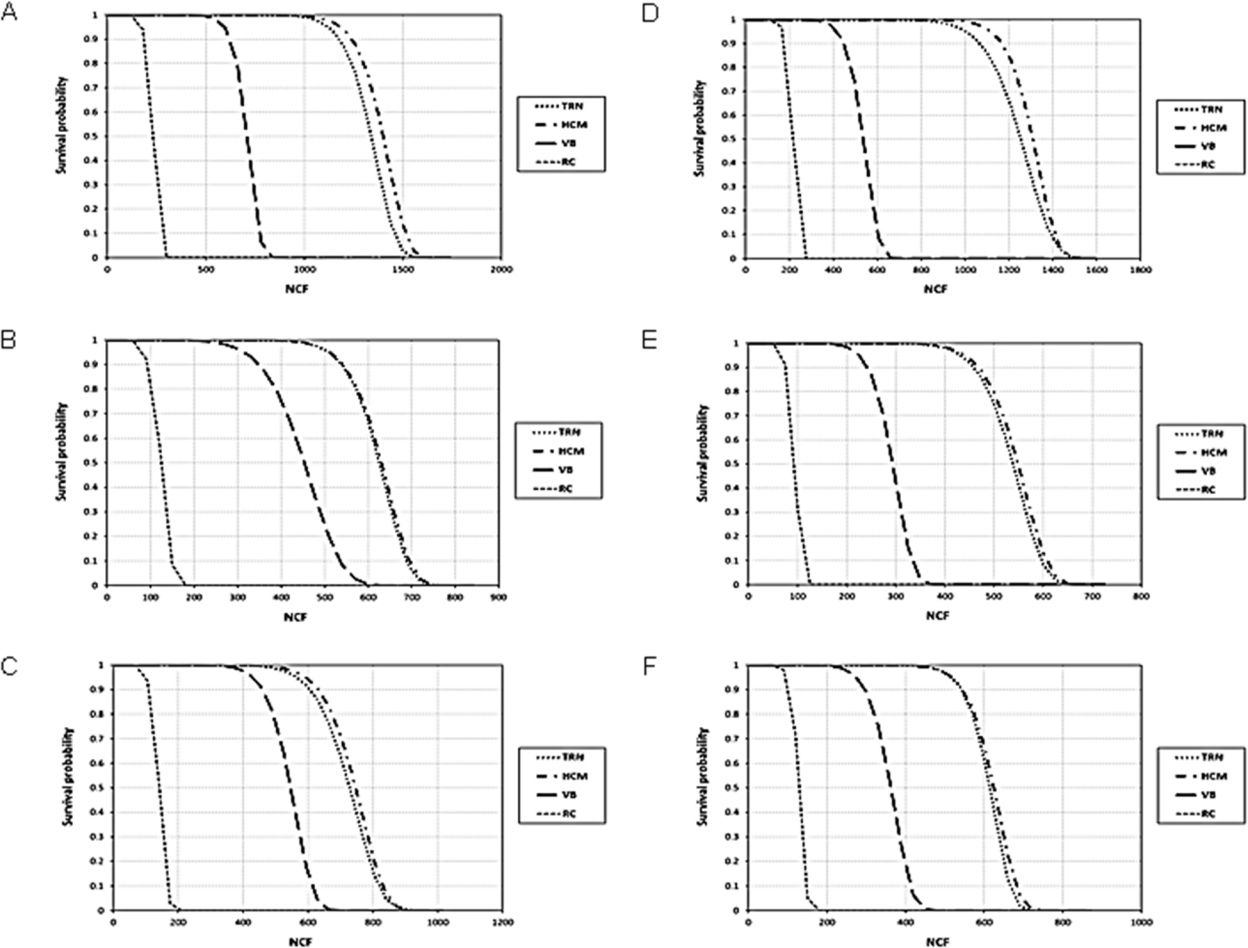
Survival probability plots for TRN, HCM, VB and RC instruments in single and double curvature canals. (**a-c**) Instruments with size 20/.04 taper for single, double apical and double coronal curvatures; respectively. (**d-f**) Instruments with size 25/.04 taper for single, double apical and double coronal curvatures; respectively

The data of Weibull modulus (m), correlation coefficient (R^2^), characteristic strength (σ_0_) and predicted cycles for 99% survival for the NCF data for each group are presented in Table 2. The cumulative probability of survival plots for the NCF data of the tested instruments are presented in Fig. 3. The instruments with size 20/.04 taper (Fig. 3a-c) revealed higher reliability than instruments with a size 25/.04 taper (Fig. 3d-f). HCM and TRN instruments showed higher reliability compared with VB and RC instruments. The predicted cycles for 99% survival of instruments tested in single curvature were higher than the instruments tested in the double curvature canal (Table 2).

**Table 2:** Weibull analysis of tested instruments

Groups	Single curvature				Double curvature							
					Apical curvature				Coronal curvature			
	Weibull modulus (m)	Correlation coefficient (R^2^)	Characteristic strength (σ_0_)	Predicted cycles for 99% survival	Weibull modulus (m)	Correlation coefficient (R^2^)	Characteristic strength (σ_0_)	Predicted cycles for 99% survival	Weibull modulus (m)	Correlation coefficient (R^2^)	Characteristic strength (σ_0_)	Predicted cycles for 99% survival
TRN												
20/.04	14.7	0.92	1375	1005	12.8	0.96	644	449	15.2	0.97	755	484
26/.04	11.7	0.84	1293	874	11.8	0.94	556	377	10.3	0.93	630	466
HCM												
20/.04	16.6	0.91	1434	1069	12.6	0.98	649	450	13.9	0.96	771	514
25/.04	15.7	0.82	1338	1014	11.9	0.97	566	385	11.3	0.91	642	461
VB												
20/.04	14.8	0.93	729	535	9.9	0.94	477	253	10.9	0.97	565	371
25/.04	10.5	0.81	555	359	7.2	0.92	305	192	9.5	0.91	377	232
RC												
20/.04	11.1	0.96	245	149	8.9	0.93	131	66	9.9	0.93	149	84
25/.04	9.1	0.93	226	147	6.7	0.91	98	59	7.7	0.88	134	82

## Discussion

Various factors can affect the fracture resistance of NiTi rotary instruments including alloy composition, manufacturing methods, cross-sectional geometry and flute design [26, 27]. Thermomechanical technology is commonly used for improving the microstructure and transformation behaviors of NiTi instruments in order to enhance the performance of instruments during root canal shaping including the cyclic fatigue resistance [28, 29]. In the present study, HCM and TRN instruments showed greater cyclic fatigue resistance than VB and RC instruments. HCM instruments are manufactured from CM heat-treated alloy that controls the instrument memory [4], which allows superior maintenance of the original canal curvature and enhanced the efficiency of the instrument in root canal preparation [9, 11]. HCM instruments are characterized by a triangular cross-sectional design displaying three cutting edges except for the instruments with size 20/.04 and 25/.04 taper, which have a square cross-section design with four blades and four flutes [30] as in the present study (Fig. 2b).

The newly developed TRN instruments are manufactured from heat-treated NiTi alloy that supposed to enhance the flexibility and fatigue resistance of the instrument [22]. TRN instruments revealed a parallelogram cross-section design (Fig. 2a) while HCM showed a square cross-section design (Fig. 2b). Both HCM and TRN instruments revealed enhanced fatigue resistance with no significant difference between them. This finding could be attributed to the manufacturing process as HCM and TRN instruments are manufactured from heat-treated NiTi alloy. The thermomechanical treatment of endodontic instruments produced instrument with different austenite finishing temperature (Af), which affects the mechanical properties especially the fatigue resistance and bending properties [31, 32]. The heat treatment of HCM instrument is based on shifting the austenite/martensite transition temperature so that a stable martensitic microstructure is obtained at body temperature [32]. Although HCM and VB instruments are manufactured from heat-treated NiTi alloy, the HCM instrument had a higher fatigue resistance. It had been reported that VB instruments had a higher degree of austenite than HCM instruments at body temperature [33]. It could be postulated that HCM and TRN instruments might be used more safely in curved canals with double curvature due to their superior fatigue resistance.

TRN instruments revealed higher fatigue resistance than VB and RC instruments. This finding could be attributed to the special heat treatment of the alloy and the design of the instruments that enhanced fatigue resistance. Heat treatment of the alloy enhances the arrangement of the crystal structure, which might improve the flexibility and strength of the NiTi instruments [10]. In addition, heat treatments of NiTi instruments during or after the manufacturing process reducing the internal stress and surface defects due to the grinding process [10].

VB instruments showed superior fatigue resistance than RC instruments. VB instrument is manufactured from NiTi Blue alloy with a reduced shape memory characteristic which improved the fatigue resistance of the instrument [2, 7, 12]. On the other hand, the RC instrument is manufactured from conventional NiTi alloy that influenced the fatigue resistance of the instrument [2, 12]. RC instruments revealed the lowest fatigue resistance among the tested instruments.

The double curvature canals (S-shaped) created more stress on NiTi rotary instruments than in single curvature canals, and consequently, the instruments fractured due to cyclic fatigue [12, 19, 29]. The S-shaped canal is one of the most challenging conditions in clinical situations during root canal instrumentation with NiTi rotary instruments [19]. In many cases, the double curvatures are not detected in conventional radiographs; consequently, the clinician should be cautious of this probability and continue carefully during root canal instrumentation [3]. In the double curvature canal, the instruments fractured first in the apical curvature followed by the coronal curvature. This finding could be attributed to the abrupt curvature in the apical area with a 2 mm radius compared with the coronal curvature with a 5 mm radius, which is in agreement with the previous studies [3, 19, 34]. There was no significant difference in the mean length of the broken fragments of tested instruments in the same curvature. The instruments fractured at or just below the centre of curvature, which is in agreement with the previous studies [3, 12, 34].

The Weibull analysis revealed that the instruments with size 20/.04 taper revealed higher reliability than instruments with size 25/.04 taper. The instruments tested in a single curvature canal revealed higher predicted cycles for 99% survival compared with instruments tested in the double curvature canal. The probability of survival was higher for HCM and TRN instruments than VB and RC instruments. RC instruments had the lowest predicted number of cycles compared with the other groups. Weibull analysis is an appropriate method to predict the survival probability of NiTi rotary instruments [24, 35].

## Conclusions

HCM and TRN had superior fatigue resistance than VB and RC in single and double curvature canals. HCM and TRN instruments were anticipated to survive with higher number of cycles than the other tested instruments. RC had the lowest fatigue resistance than other instruments.

## Data Availability

The datasets used and/or analysed during the current study are available from the corresponding author on reasonable request.

## Abbreviations

Af: Austenite finishing temperature
CM: Controlled memory
HCM: HyFlex CM
m: Weibull modulus
NCF: Number of cycles to failure
NiTi: Nickel-titanium
R^2^: Correlation coefficient
RC: RaCe
SEM: Scanning electron microscope
TRN: TruNatomy
VB: Vortex Blue
σ_0_: Characteristic strength
